# Modulation of the gut microbiota as a novel strategy to prevent anastomotic leak after colorectal surgery: Systematic scoping review

**DOI:** 10.1111/codi.70472

**Published:** 2026-05-04

**Authors:** Jack A. Helliwell, Peter Sciberras, Alexios Dosis, Joshua Burke, Caroline H. Chilton, Henry M. Wood, David G. Jayne

**Affiliations:** ^1^ Leeds Institute of Medical Research University of Leeds Leeds UK

**Keywords:** anastomotic leak, colorectal surgery, gut microbiota, microbiome modulation

## Abstract

**Background:**

Anastomotic leak (AL) remains a major source of morbidity following colorectal surgery. Increasing evidence implicates the gut microbiome in the pathogenesis of AL, with certain microbial species disrupting tissue repair through collagen degradation. Perioperative modulation of the microbiome may offer a novel strategy to improve anastomotic healing. This scoping review aimed to map available evidence on microbiome‐targeted interventions, synthesise mechanistic insights, and identify translation gaps in relation to anastomotic outcomes.

**Methods:**

A systematic scoping review was performed. MEDLINE, Embase and Cochrane Central Registry of Controlled Trials databases were searched from database inception to 5th August 2025. Studies were eligible if they investigated perioperative interventions that modulated the gut microbiome and evaluated anastomotic healing or leak rates. Both clinical and preclinical studies were included. A narrative synthesis was performed by charting key findings.

**Results:**

Of 4209 records screened, 27 studies met the inclusion criteria: 9 clinical and 18 preclinical. Interventions included bowel preparation, probiotics, synbiotics, arginine/omega‐3 supplementation, dietary modification, faecal microbiota transplantation (FMT), phosphate, tranexamic acid, morphine and infliximab. Among clinical studies, only oral antibiotics combined with mechanical bowel preparation were associated with a significant reduction in leak rates. Preclinical studies showed interventions such as high‐fibre diets, FMT, rectal tranexamic acid and phosphate supplementation improved anastomotic healing via enhanced microbial diversity, suppression of pathogenic organisms, or inhibition of collagenolytic activity.

**Conclusion:**

This review highlights a range of microbiome‐targeted interventions with potential to reduce AL. While clinical evidence remains limited, several preclinical strategies demonstrate promise and warrant evaluation in early‐phase human trials.

## INTRODUCTION

Anastomotic leak (AL) remains one of the most feared complications following colorectal surgery, associated with considerable morbidity and mortality. Clinical consequences include intra‐abdominal abscess, faecal peritonitis, sepsis, and multiorgan failure. Despite advances in surgical technique and perioperative care, the incidence of AL has remained largely unchanged, affecting approximately 10%–15% of patients [1, 2].

Emerging evidence implicates the gut microbiome as a contributing factor in anastomotic failure. Certain commensal organisms, notably *Enterococcus faecalis* and *Pseudomonas aeruginosa*, have been shown to colonise the anastomotic site, evade standard perioperative antibiotics, and secrete collagen‐degrading enzymes that impair the structural integrity of the healing tissue [3]. Preclinical studies suggest that surgical stressors–including ischaemia, malnutrition, and radiation–can disrupt the gut microbiota, resulting in dysbiosis [4]. This is characterised by a reduction of protective bacterial species and an increase in pathogenic bacteria capable of collagenase production and activation of host matrix metalloproteinases [5].

**FIGURE 1:**
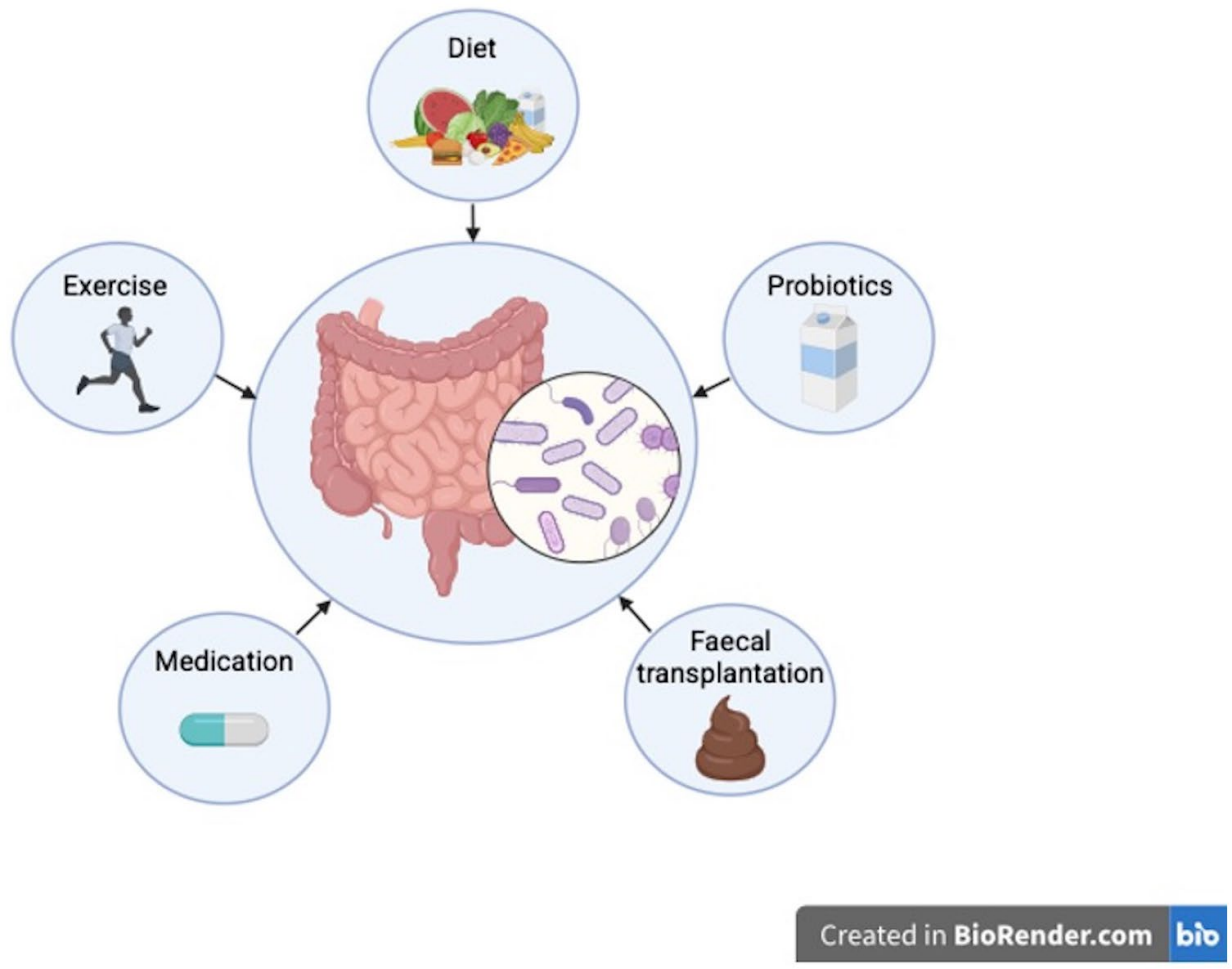
Factors affecting the gut microbiome. Schematic representation of key factors proposed to influence the composition and function of the gut microbiome. These include dietary composition, probiotic supplementation, faecal microbiota transplantation, perioperative medications and physical activity, each of which may alter microbial diversity, abundance of specific taxa, and functional capacity.

Importantly, the gut microbiome is dynamic and modifiable by a range of perioperative factors, including diet, antibiotics and other medications (Figure 1). As such, targeted modulation of the microbiome represents a potential strategy to enhance anastomotic healing and reduce the risk of leak. Potential approaches may include increasing the abundance of beneficial microbial species, reducing pathogenic organisms, or suppressing virulence factors such as collagenolytic enzymes.

This scoping review aimed to map the available evidence on perioperative interventions that influence the gut microbiome and may affect anastomotic healing following colorectal surgery. Clinical studies were assessed first, followed by relevant preclinical data, with a focus on identifying mechanistic insights and translational gaps to inform future research.

## METHODS

### Study design

A systematic scoping review was performed according to a predefined protocol to identify and map the available evidence. Scoping reviews are not eligible for registration on the PROSPERO database of systematic reviews. Considering the anticipated high degree of variability among included studies, a quantitative synthesis of the outcomes was not planned. The purpose of this review was to map the breadth and nature of the available evidence, rather than to estimate comparative effectiveness between interventions. The manuscript adheres to the PRISMA Extension for Scoping Reviews guidelines [6].

### Search strategy

A search strategy was developed by combining keywords related to the microbiome, AL, and colorectal surgery, using Boolean operators (Table S1). A systematic search of MEDLINE (via OVID), EMBASE (via OVID), and Cochrane Central Registry of Controlled Trials (via OVID) databases was performed for articles published between the inception of the databases and 5th August 2025. Three independent investigators (JAH, PS, AD) reviewed titles, abstracts, and full‐text papers. Disagreements were resolved through discussion and consultation with the authorship team until consensus was reached.

### Eligibility criteria

Studies were eligible for inclusion if they provided mechanistic insight into microbiome changes associated with a perioperative intervention and assessed the intervention's impact on anastomotic healing and/or leak rates. All original study types were eligible, including preclinical studies (such as experimental animal studies) and clinical studies involving human participants. Grey literature, such as conference extracts, were also eligible for inclusion. Articles published in languages other than English were excluded.

### Definitions

A perioperative intervention was defined as any treatment provided before, during, or after surgery, without predefined restrictions on the timing or route of administration. Colorectal surgery was defined as surgery on the colon or rectum, irrespective of surgical approach or clinical indication. The gut microbiome was defined as the community of microorganisms present in the gastrointestinal tract.

### Study outcomes

Primary variables of interest included characteristics of each perioperative intervention (type, route, and timing), changes in microbiome composition (diversity metrics, relative abundance of specific taxa or species), and measures of anastomotic healing or leak.

Additional data extracted included study characteristics such as country of origin, journal, population studied (including animal model and anastomotic site), and microbiome assessment methods (sample type, collection timepoints, and sequencing or analytical methods).

### Data extraction and charting

Data were extracted from included studies by a single investigator (JAH) and verified by a second investigator (PS). This was done using a semi‐structured data extraction spreadsheet in Microsoft Excel.

### Statistical analysis

Data were analysed descriptively using averages, proportions and rates, where applicable. No quantitative syntheses of outcomes or assessment of study quality were conducted. Instead, a narrative synthesis of collected data is provided.

## RESULTS

### Study characteristics

**FIGURE 2:**
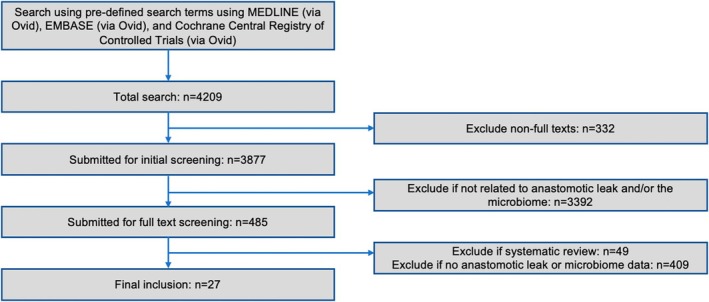
Prisma flow diagram of study eligibility.

A total of 4209 records were screened, with 27 studies meeting the eligibility criteria and included in the analysis (Figure 2).

Nine clinical studies were conducted across Europe and Asia, with sample sizes ranging from 40 to 455 participants (Table S2) [7, 8, 9, 10, 11, 12, 13, 14, 15]. Most included mixed populations undergoing left‐ and right‐sided colorectal resections, with some focusing specifically on patients with malignancy. Microbiome sampling was primarily performed via stool or rectal swabs and predominantly analysed using 16S rRNA sequencing. The primary outcome varied among these studies, with AL the primary outcome of one.

Eighteen preclinical studies were performed in murine models, predominantly originating from North America (Table S3) [5, 16, 17, 18, 19, 20, 21, 22, 23, 24, 25, 26, 27, 28, 29, 30, 31, 32]. The majority employed a distal colonic end‐to‐end anastomosis model, with some incorporating high‐risk modifications such as pelvic irradiation, ischaemia, or inoculation with pathogenic organisms. Microbiome sampling typically involved stool analysis, though some studies also examined anastomotic tissue. Anastomotic healing was primarily assessed through macroscopic grading, supplemented by histological or mechanical analysis in select studies.

### Bowel preparation

Three clinical studies and one preclinical study investigated preoperative bowel preparation strategies, including mechanical bowel preparation (MBP), oral antibiotics, or selective decontamination of the digestive tract (SDD) (Tables 1 and 2) [7, 8, 9, 16].

**TABLE 1:** Results of included clinical studies.

study (year)	Features of intervention			Microbiome findings	Anastomotic leak (AL) data
	Type	Route (dose)	Timing (duration)		
Zukauskaite (2024) [7]	Mechanical bowel preparation (macrogol 4000, 4 litres)	Oral	Pre‐op (1 day)	No difference in beta‐diversity. Similar dysbiosis in both groups.	No significant difference. MBP: 0/20 Enema: 1/20
Sadahiro (2014) [8]	Antibiotics (kanamycin sulfate, metronidazole)	Oral	Pre‐op (1 day)	↓ Bacteroides and Escherichia/Shigella. ↑ Enterococcus in all groups	Significantly lower AL rate Antibiotics: 1/155 Probiotics: 12/155 *
Reuvers (2023) [9]	Selective decontamination of digestive tract (amphotericin B, colistin, tobramycin)	Oral	Pre‐op (4 days); Post‐op (4 days)	↓ Proteobacteria, Enterobacteriaceae and E. coli	No significant difference. SDD: 14/226 Control: 22/229
Park (2020) [10]	Probiotic supplementation (Bifidobacterium animalis, Lactobacillus casei, Lactobacillus plantarum)	Oral (2 × 10^8^ cfu/2 g)	Pre‐op (7 days); Post‐op (21 days)	↑ Bacteria with probiotic effects. ↓ Colon cancer associated bacteria	No significant difference. Probiotics: 0/29 Placebo: 1/31
Liu (2010) [11]	Probiotic supplementation (Lactobacillus plantarum, Lactobacillus acidophilus, Bifidobacterium longum)	Oral (2.6 × 10^14^ cfu/2 g)	Pre‐op (6 days); Post‐op (10 days)	↑ Bifidobacteria and Lactobacilli. ↓ Entero‐bacteriaceae, Pseudomonas, Candida	No significant difference Probiotics: 0/50 Placebo: 0/50
Mizuta (2016) [12]	Probiotic supplementation (Bifidobacterium longum)	Oral (5 × 10^10^ cfu/2 g)	Pre‐op (7–14 days); Post‐op (14 days)	↑ Actinobacteria and Firmicutes	No significant difference Probiotics: 3/31 Control: 5/31
Komatsu (2015) [13]	Synbiotic supplementation (Lactobacillus casei, galacto‐oligosaccharide, Bifidobacterium breve)	Oral (5 × 10^10^ cfu/ 180 mL)	Pre‐op (7–11 days), Post‐op (2–7 days)	↓ Enterobacteriacae and Pseudomonas	No significant difference. Synbiotics: 12/173 Control: 12/206
Reddy (2007) [14]	Synbiotic supplementation (Lactobacillus acidophilus, Lactobacillus bulgaris, Bifidobacterium lactis, Streptococcus thermophilus)	Oral (4 × 10^9^ cfu)	Pre‐op	↓ Enterobacteriaceae	No significant difference 2/88 leaks in study population
Lee (2023) [15]	Diet supplemented with arginine and omega‐3 fatty acids	Oral (400 mL/day)	Pre‐op (7 days)	No significant difference in microbiome	No significant difference. 0 leaks in study population

*Note*: * indicates *p* value < 0.05.

Abbreviations: AL, anastomotic leak; CFU, colony forming units; MBP, mechanical bowel preparation.

**TABLE 2:** Results of included preclinical studies.

Study (year)	Intervention			Microbiome findings	Anastomotic leak (AL) data
	Type	Route	Timing (duration)		
Boatman (2024) [16]	High‐fat/ High‐sugar diet	Oral	Pre‐op (10–12 weeks)	↓ Alpha‐diversity.	↔ AL: 31/71 versus 21/71 versus (*p* = 0.08)
	Mechanical bowel preparation and oral antibiotics (neomycin, metronidazole)	Oral	Pre‐op (1 day)	No difference in alpha‐diversity No difference in butyrate, propionate, and acetate	↔ AL: 17/35 versus 11/36 (*p* = 0.09)
Guo (2024) [17]	High‐fat/ Low‐fibre diet	Oral	Pre‐op (6 weeks)	↓ Alpha‐diversity.	↔ AHS: 1.67 versus 1.75 (*n* = 26, *p* = 0.81)
	Subcutaneous antibiotic (enrofloxacin)	Subcut	Pre‐op (1 day)	↓ Enterococcus.	↓ AHS: 1.33 versus 2.08 (*n* = 26, *p* = 0.02) *
Hyoju (2021) [18]	Low‐fat/High‐fibre dietary prehabilitation	Oral	Pre‐op (2, 4 or 6 days)	↑ Alpha‐diversity. ↑ Bacteriodes. ↓ collagenolytic Enterococcus	↓ AHS: 2.0 versus 2.8 (*n* = 2, *p* = 0.025) *
Liu (2009) [19]	Probiotic supplementation (Lactobacillus plantarum)	Oral	Pre‐op (15, 18, or 22 days)	No difference in bacterial microflora.	↑ Collagen conc.: 13 versus 10 microgram/mg (*n* = 24, *p* < 0.05) *
Casthilo (2023) [20]	Probiotic supplementation (Lactobacillus paracasei, Bifidobacterium lactis, Lactobacillus rhamnosus, Lactobacillus acidophilus)	Oral	Pre‐op (7 days), Post‐op (5 days)	↓ Clostridium, Pravotella, Streptococcus, and Enterococcus. ↑ Lactobacillus, Bacterioides, Blauta and Dorea	↑ Traction force: 1.5 versus 1.2 N (*n* = 36; *p* = 0.025) *
Hajjar (2021) [21]	Prebiotic supplementation (inulin, galacto‐oligosaccharides)	Oral	Pre‐op (2 weeks)	↑ butyrate ↓ collagenolytic enzyme activity at anastomosis	↓ AHS: 1.0 (inulin) versus 1.3 (GOO) versus 2.3 (cellulose) (*n* = 45; *p* < 0.05) *
Mocanu (2021) [22]	Prebiotic supplementation (tributyrin)	Oral	Pre‐op (1 week), Post‐op (1 week)	↑ anaerobic and aerotolerant organisms	↔ HIS: 1.5 versus 1.5 (*n* = 24)
Wiege‐rinck (2018) [23]	Phosphate supplementation (Poly‐phosphorylated polymer ABA‐PEG20k‐Pi20)	Oral	Pre‐op (7 days)	↓ collagenolytic activity of E. faecalis ↓ Enterococcus at anastomotic site	↓ AHS: 2.0 versus 2.7 (*n* = 30; *p* = 0.025) *
Hyoju (2019) [24]	Phosphate supplementation (Polyphosphate compound PPI‐6)	Oral	Pre‐op (11 days)	↓ collagenolytic activity, biofilm production and motility of S. marcescens, P. aeruginosa.	↓ AHS: 1.2 versus 2.3 (*n* = 10; *p* < 0.05) *
Olivas (2012) [25]	Phosphate enema (Polyethylene glycol polymer with phosphate buffer (PEG/Pi) enema)	Rectal	Intra‐op	↓ transformation of P. aeruginosa to tissue destructive P2 phenotype	↓ AL: 10% versus 70% (*n* = 10, *p* < 0.01) *
Jacobson (2020) [26]	Tranexamic acid enema	Rectal	Post‐op (3 days)	E. faecalis‐ mediated plasminogen activation leads to supraphysiological collagen degradation	↓ AL: 0/10 versus 6/10 (*p* < 0.05) *
Jacobson (2021) [27]	Tranexamic acid enema	Rectal	Post‐op (3 days)	↓ Collagenolytic bacterial counts and plasminogen deposition	↓ AHS: 1.4 versus 1.7 (*n* = 20, *p* < 0.05) *
Shogan (2016) [5]	Antibiotic enema (ciprofloxacin, metronidazole, neomycin)	Rectal	Intra‐op, Post‐op (2 days)	↓ Collagenase‐producing E. faecalis. ↓ Bacterial collagenase production	↓ AL: 0% versus 60% (*n* = 10, *p* < 0.01) *
Jin (2022) [28]	Faecal microbiota transplantation (stool from patient who did not leak)	Oral	Pre‐op (1 week)	Microbiome paralleled engrafted faeces ↑ Escherichia/Shigella in leak ↑ Lactobacillus in no leak	↑ collagen, epithelial–mesenchymal transformation, and TGF‐beta signalling
Boatman (2023) [29]	High‐fat/ High‐sugar diet	Oral	Pre‐op (8–10 weeks)	↓ Alpha‐diversity	↔ AL: 5/15 versus 6/15 (*p* = 0.71)
	Faecal microbiota transplantation (stool from mice fed lean diet)	Oral	Pre‐op (1 day)	↑ Alpha‐diversity	↓ AL: 0/13 versus 5/15 (*p* = 0.04) *
Hajjar (2023) [30]	Faecal microbiota transplantation (stool from patient who did not leak)	Oral	Pre‐op (Not stated)	Parabacteroides goldensteinii associated with improved healing Alistepes onderdonkii associated with higher leak rates	↓ AHS: 1.57 versus 2.85 (*n* = 8; *p* = 0.0023) *
Shakh‐sheer (2016) [31]	Morphine	Subcut	Post‐op (6 days)	↑ collagenase and biofilm production by E. faecalis	↑ AHS: 1.35 versus 0.1 (*n* = 250, *p* < 0.05) *
Gaines (2020) [32]	Infliximab	Intra‐ peritoneal	Pre‐op (8 weeks)	↑ Turcibacter. No increase in perianastomotic collagenase‐producing bacteria	↔ AHS: 1.75 versus 1.75 (*n* = 10, *p* > 0.05)

*Note*: AHS is a validated macroscopic measure of anastomotic healing, where higher scores indicate poorer healing. Score 0 = normal; 1 = flimsy adhesions; 2 = dense adhesion without abscess or intraperitoneal contamination; 3 = dense adhesions with gross abscess at the anastomotic site; 4 = gross leak with peritoneal contamination and a visible anastomotic dehiscence. HIS is a validated score which evaluates enterocyte injury, epithelial hyperplasia, lamina propria, lymphocyte, and lamina propria neutrophils. * indicates *p* value < 0.05.

Abbreviations: AL, anastomotic leak; AHS, anastomotic healing score; HIS, histological injury score.

A pilot RCT (*n* = 40) compared oral MBP (macrogol 4000, 4 litres) with rectal enema preparation [7]. No significant difference in beta‐diversity was observed between groups, and AL occurred in only one participant across the study population (0/20 patients in the MBP group vs. 1/20 in the enema group).

A larger clinical study (*n* = 310) examined the use of oral antibiotics (kanamycin and metronidazole) in combination with MBP [8]. Participants who received oral antibiotics demonstrated a significantly lower AL rate (1/155 vs. 12/155 in the control group; *p* < 0.05). However, no significant differences in overall microbiome composition were observed, and therefore the mechanism underlying this effect remains uncertain.

One preclinical study also investigated the effect of MBP and oral antibiotics (neomycin and metronidazole) using a mouse model [16]. Here, oral antibiotics were not associated with changes in alpha‐diversity or short‐chain fatty acid concentrations (butyrate, propionate, acetate), nor with a significantly lower incidence of AL (17/35 vs. 11/36; *p* = 0.09).

In a third clinical study (*n* = 452), SDD with amphotericin B, colistin, and tobramycin administered orally for 4 days preoperatively and 4 days postoperatively was evaluated [9]. The relative abundance of *Proteobacteria*, *Enterobacteriaceae*, and *E. coli* was significantly lower in the SDD group. While no significant difference in AL was reported (14/226 vs. 22/229; OR 0.63; 95% CI: 0.31–1.29), a reduced incidence of infective complications was observed in the SDD group.

### Dietary interventions

Four preclinical studies evaluated the effects of dietary composition on the gut microbiome and anastomotic healing (Table 2) [16, 17, 18, 29].

Three studies demonstrated that a high‐fat, high‐sugar, low‐fibre diet administered for 6–12 weeks before surgery was associated with reduced alpha‐diversity, although this did not significantly affect anastomotic healing or leak rates in animal models [16, 17, 29]. Specifically, in Boatman (2024), AL occurred in 31/74 animals on the high‐fat diet versus 21/71 controls (*p* = 0.08). In Boatman (2023), leak rates were 5/15 versus 6/15 (*p* = 0.71), and Guo (2024) reported no difference in mean anastomotic healing score (1.67 versus 1.75; *n* = 26; *p* = 0.81).

One study showed that short‐term dietary prehabilitation with a high‐fibre diet was able to reverse the adverse effects of a high‐fat diet [18]. Mice who received a high‐fibre diet for 2–6 days before surgery showed restored microbial diversity and significantly improved anastomotic healing score (AHS: 2.0 vs. 2.8; *n* = 2; *p* = 0.025).

### Probiotic supplementation

Three clinical studies and two preclinical studies assessed perioperative probiotic supplementation, typically involving strains of *Lactobacillus* and/or *Bifidobacterium* (Tables 1 and 2) [10, 11, 12, 19, 20].

Probiotics were generally administered orally for 6–22 days preoperatively and continued for 10–21 days postoperatively, with dosages ranging from 2 × 10^8^ to 2.6 × 10^14^ colony forming units. Reported microbiome changes included increased abundance of *Actinobacteria*, *Lactobacillus*, and *Bifidobacterium*, along with reductions in *Pseudomonas*, *Candida*, and other pathogenic organisms.

None of the clinical studies demonstrated a significant reduction in AL rates., although all appeared underpowered, with samples sizes ranging from 60 to 100 participants (Table 1) [10, 11, 12].

Among the two preclinical studies, probiotic supplementation was associated with improved anastomotic collagen concentration (13 vs. 10 μg/mg; *n* = 24; *p* < 0.05) [19] and increased tensile strength of the anastomosis (1.5 vs. 1.2 N; *n* = 36; *p* = 0.025) [20].

### Synbiotic and prebiotic supplementation

Two clinical studies evaluated synbiotic supplementation, combining probiotics and prebiotics [13, 14]. Both studies reported a reduction in *Enterobacteriaceae* abundance; however, neither demonstrated a significant decrease in AL rates (Table 1). In Komatsu (2015), leaks occurred in 12/173 patients receiving synbiotics versus 12/206 controls. In Reddy (2007), only two leaks were reported among 88 patients overall.

In preclinical models, two studies assessed prebiotic supplementation alone, utilising inulin, galactooligosaccharides (GOS), and/or tributyrin [21, 22]. Supplementation with inulin and GOS was associated with increased populations of anaerobic and aerotolerant organisms, elevated butyrate levels and reduced collagenolytic enzyme activity at the anastomotic site [21]. These changes corresponded with improved anastomotic healing score for both inulin (AHS: 1.0 vs. 2.3; *n* = 30; *p* < 0.001) and GOS (AHS: 1.3 vs. 2.3; *n* = 30; *p* < 0.05) as well as enhanced mucosal continuity. Conversely, tributyrin supplementation alone did not significantly alter histological injury scores (1.5 vs. 1.5; *n* = 24) but was linked to reduced intestinal inflammation (Table 2) [22].

### Arginine and omega‐3 fatty acid supplementation (Immunonutrition)

One clinical study investigated the effect of perioperative arginine and omega‐3 fatty acid supplementation (Immunonutrition), administered orally for 7 days preoperatively [15]. No significant differences in gut microbiota composition were observed between the intervention and control groups. AL did not occur in any of the 176 patients included (Table 1).

### Phosphate supplementation

Two preclinical studies evaluated the use of oral phosphate supplementation administered for 7–11 days before surgery (Table 2) [23, 24].

In both, phosphate supplementation was associated with reduced collagenolytic activity of *Enterococcus faecalis*, *Pseudomonas aeruginosa*, and *Serratia marcescens*. This effect was accompanied by improved anastomotic healing, as reflected by lower mean anastomotic healing scores: Wiegerinck (2018), 2.0 versus 2.7 (*n* = 30, *p* = 0.025), and Hyoju (2019), 1.2 versus 2.3 (*n* = 10, *p* < 0.05).

### Rectal therapies

Four preclinical studies investigated rectally administered agents, including phosphate enema [25], tranexamic acid enema [26, 27], and antibiotic enema [5] (Table 2).

Intraoperative rectal administration of phosphate was associated with reduced colonisation by collagenase‐producing *Enterococcus faecalis* and prevention of anastomotic leak (AL rate: 10% vs. 70%; *n* = 10; *p* < 0.01) [25].

Rectal tranexamic acid administered intraoperatively and for 3 days postoperatively was associated with reduced bacterial collagenase production and improved anastomotic healing (AHS: 1.4 vs. 1.7; *n* = 20; *p* < 0.05) [27]. Specifically, tranexamic acid inhibited plasminogen activation by *Enterococcus faecalis*, leading to decreased collagen degradation [26].

An antibiotic enema containing ciprofloxacin, metronidazole, and neomycin, administered intraoperatively and for 2 days postoperatively, was associated with reduced anastomotic colonisation by *Enterococcus faecalis* and prevention of AL (0% vs. 60%; *n* = 10; *p* < 0.01) in a devascularised anastomotic model [5].

### Faecal transplantation

Three preclinical studies investigated the use of faecal microbiota transplantation (FMT) [28, 29, 30].

Two studies administered faecal material from human donors without AL to recipient mice and compared outcomes with transplants from a donor who had experienced AL [28, 30]. In both studies, recipient microbiota profiles closely resembled those of the respective donors. Notably, *Lactobacillus* was more abundant in the non‐leak group, with higher proportions of *Escherichia/Shigella* in the AL group. FMT from non‐leak donors was consistently associated with improved anastomotic healing (Table 2). Jin (2022) reported increased collagen deposition, enhanced epithelial–mesenchymal transformation, and upregulation of TGF‐beta signalling, while Hajjar (2023) demonstrated improved anastomotic healing, as evidenced by a lower anastomotic healing score (1.57 vs. 2.85; *n* = 8; *p* = 0.0023).

In the third study, faecal material for transplant was obtained from mice fed a lean diet [29]. This intervention increased microbial alpha‐diversity and was associated with a significantly lower AL rate compared with controls (0/13 vs. 5/15; *p* < 0.04).

### Perioperative medications

Two preclinical studies evaluated medications encountered during the perioperative period, including morphine [31] and infliximab [32].

Systemic administration of morphine for 6 days postoperatively was associated with increased collagenase activity and adherence capacity of *Enterococcus faecalis*, parameters that were in turn associated with reduced anastomotic healing (AHS: 1.35 vs. 0.1; *n* = 250; *p* < 0.05) [31].

Conversely, infliximab administered intraperitoneally for 8 weeks before surgery did not demonstrably impair anastomotic healing (AHS: 1.75 vs. 1.75; *n* = 10; *p* > 0.05), although it altered the anastomotic microbiome with emergence of *Turcibacter* [32].

## DISCUSSION

The present review provides a comprehensive overview of perioperative interventions aimed at modulating the microbiome to prevent AL. Despite increasing evidence implicating the gut microbiome in the pathogenesis of AL, clinical research in this area remains limited. Among the clinical studies identified, only MBP combined with oral antibiotics was associated with a statistically significant reduction in leak rate, although no corresponding differences in microbiome composition were demonstrated to explain this effect. This finding is consistent with the broader literature supporting the use of oral antibiotics during bowel preparation [33, 34].

In contrast, preclinical studies identified a range of microbiome‐modulating strategies with promising therapeutic potential. These included dietary prehabilitation, FMT, phosphate supplementation, rectally administered tranexamic acid and antibiotics, as well as probiotic and prebiotic therapies. Across various animal models, these interventions reproducibly altered microbial composition and improved histological, mechanical, or macroscopic indicators of anastomotic healing. Additionally, the detrimental effects of morphine on both the microbiome and anastomotic healing were noted, reinforcing the case for opioid‐sparing analgesic strategies in the perioperative setting.

Mechanistically, these interventions appear to act through several distinct yet interrelated pathways. Strategies such as dietary prehabilitation, prebiotics, and probiotics enhanced microbial diversity and increased the relative abundance of commensal, health‐promoting bacteria. FMT facilitated improved anastomotic healing by transferring key features of a healthy microbiome–namely, high diversity, a predominance of beneficial taxa, and suppression of pathogenic organisms. Other interventions acted by directly targeting microbial pathogens. Both systemic and rectal antibiotics reduced the local burden of harmful bacteria at the anastomotic site, including species known to produce collagen‐degrading enzymes that compromise tissue integrity.

Phosphate and tranexamic acid operated through more specific molecular mechanisms. Phosphate influenced bacterial behaviour via microbial phosphate‐sensing pathways [35]. Under normal phosphate‐rich conditions–typical of a healthy mucus layer in the bowel–bacteria remain in a non‐invasive state. However, surgical stress can disrupt this mucus layer, resulting in phosphate depletion. In response, pathogenic species may upregulate collagenase production to invade host tissues. Supplementation with phosphate appears to suppress this virulence response [35]. Tranexamic acid, on the other hand, acted by inhibiting the host fibrinolytic system, particularly plasminogen activation, a pathway exploited by certain bacteria to enhance collagenase activity [26].

While the breadth of strategies explored is encouraging, important limitations of the included studies must be acknowledged. Many of the clinical trials identified were underpowered, restricting their ability to detect potential differences in leak rates. Heterogeneity in study design, intervention type, and dosing further complicates interpretation, particularly in clinical trials evaluating probiotic and synbiotic supplementation. Even where reductions in leak rates were reported, as with oral antibiotics combined with MBP, these were not accompanied by measurable shifts in microbiome composition, raising questions about the assumed mechanisms of benefit.

Preclinical studies, while providing mechanistic insight, shared similar challenges. Small sample sizes were common, and many relied on surrogate markers of leak rather than AL itself. These surrogate end‐points, including anastomotic healing scores and tensile strength, may not directly correlate with clinically meaningful outcomes such as leak rates. In addition, some models employed interventions in ways that differ from typical clinical practice (e.g. intraperitoneal infliximab), limiting external validity. Murine models also differ substantially from human colorectal surgery in terms of immune response and baseline microbiome composition, which may restrict comparability. Moreover, interventions that are feasible in animal models, such as rectal antibiotics, may prove less acceptable, scalable, or tolerable in patients, while effective dosing and safety profiles remain undefined. The human perioperative setting also introduces greater heterogeneity, including variation in comorbidities, nutrition, and systemic antibiotics, all of which may influence the efficacy of microbiome‐targeted therapies. Together, these factors highlight the risk of overinterpreting preliminary preclinical findings and underscore the need for cautious stepwise clinical evaluation.

Despite their methodological limitations, preclinical studies collectively provide encouraging signals, yet translating these into clinical benefit remains a significant challenge. Rigorous early‐phase human trials are essential to assess safety, feasibility, and early signals of efficacy. Such studies must carefully consider the timing, duration, and delivery of interventions, as well as potential interactions with standard perioperative care. For example, the efficacy of FMT or probiotics may be compromised if administered in close proximity to systemic antibiotics. Rectally delivered interventions–such as antibiotics or tranexamic acid–may represent a novel, site‐specific strategy for protecting high‐risk anastomoses (e.g. after anterior resection). However, their clinical acceptability, ease of administration, and alignment with patient preferences will require co‐development with input from both patients and healthcare professionals. Importantly, microbiome profiling should be embedded within these early‐phase studies to provide mechanistic insights, identify microbial signatures of treatment response, and guide the selection of the most promising candidate interventions for further evaluation in large‐scale, definitive trials. Given the diversity of potential strategies in this space, future progress may be best supported by a platform trial design. Such adaptive frameworks would allow multiple microbiome‐targeted interventions to be tested in parallel, discontinuing those without signals of benefit while rapidly advancing the most promising candidates to definitive evaluation. This approach could accelerate translation while making more efficient use of limited patient populations.

A key strength of this review lies in its broad inclusion of both clinical and preclinical evidence. To the best of our knowledge, this is the first synthesis to integrate data across these domains in the context of microbiome‐targeted strategies for AL. This broad perspective was crucial for mapping the translation landscape and identifying avenues for future investigation. Nevertheless, limitations remain. The heterogeneity of the included studies, such as variations in study design, sample sizes, and outcomes measures, makes it challenging to draw meaningful comparisons between studies. Accepting this heterogeneity, a narrative synthesis represents the most feasible synthesis of data, particularly since the aim of the study was to describe previous evidence and identify opportunities for future clinical investigation. In addition, the requirement for studies to report both microbiome data and anastomotic outcomes may have excluded clinically relevant studies reporting leak rates alone. Although this was intentional to explore mechanistic links, it may limit generalisability and underrepresent the broader clinical evidence base.

In conclusion, although clinical evidence remains limited, this review identifies a promising pipeline of microbiome‐targeting strategies with potential to improve anastomotic healing. The development of innovative trial designs, such as early‐phase adaptive platform studies, may be key to efficiently identifying which of these strategies should progress to definitive testing. With careful evaluation and meaningful stakeholder engagement, several of these interventions could progress towards clinical application. This may offer a novel, biologically targeted approach to reducing AL–a complication that continues to resist conventional preventative strategies.

## AUTHOR CONTRIBUTIONS

**Alexios Dosis:** Investigation; validation; writing – review and editing; data curation. **Caroline H. Chilton:** Conceptualization; writing – review and editing; supervision. **Jack A. Helliwell:** Conceptualization; investigation; writing – original draft; methodology; validation; visualization; writing – review and editing; formal analysis; project administration; data curation. **David G. Jayne:** Conceptualization; writing – review and editing; supervision. **Peter Sciberras:** Investigation; validation; writing – review and editing; data curation. **Joshua Burke:** Investigation; writing – review and editing. **Henry M. Wood:** Conceptualization; writing – review and editing; supervision.

## FUNDING INFORMATION

No sources of funding were received for this study. JAH is supported by NIHR303267. CHC and HMW are supported in part by NIHR203331. DGJ is supported in part by NIHR213331 and NIHR205280. The views expressed are those of the authors and not necessarily those of the NHS, the NIHR, or the Department of Health and Social Care.

## CONFLICT OF INTEREST STATEMENT

The authors declare no conflict of interest.

## ETHICS STATEMENT

Ethical approval was not required for this study as it is a systematic scoping review of previously published literature.

## PATIENT CONSENT STATEMENT

Patient consent was not required for this study as no individual patient data were collected.

## PERMISSION TO REPRODUCE MATERIAL FROM OTHER SOURCES

No previously published material requiring permission has been reproduced in this manuscript.

## Supporting information


Table S1.
**Table S2**.
**Table S3**.

Data S1.

## Data Availability

The data that support the findings of this study are available from the corresponding author upon reasonable request.
