# Supplementary material for: Modulation of the gut microbiota as a novel strategy to prevent anastomotic leak after colorectal surgery: Systematic scoping review
Source: Colorectal Dis. 2026 May 4;28:e70472. doi: 10.1111/codi.70472 (PMC13139701; doi:10.1111/codi.70472)
Supplement: Supplementary file 1 — Table S1. Table S2. Table S3. [file CODI-28-0-s001.docx]

| 1 | Microbiome |  |
| --- | --- | --- |
| 2 | Microbiota |  |
| 3 | Microflora |  |
| 4 | Collagenolytic |  |
| 5 | Collagenase |  |
| 6 | Collagenolysis |  |
| 7 | Enterococcus faecalis |  |
| 8 | Pseudomonas aeruginosa |  |
| 9 | Serratia marcescens |  |
| 10 | Butyrate |  |
| 11 | 1 OR 2 OR 3 OR 4 OR 5 OR 6 OR 7 OR 8 OR 9 OR 10 |  |
| 12 | Anastomosis |  |
| 13 | Anastomoses |  |
| 14 | 12 OR 13 |  |
| 15 | Anastomotic |  |
| 16 | Leak |  |
| 17 | Healing |  |
| 18 | Dehiscence |  |
| 19 | Insufficiency |  |
| 20 | Failure |  |
| 21 | 16 OR 17 OR 18 OR 19 OR 20 |  |
| 22 | 15 AND 21 |  |
| 23 | 14 OR 22 |  |
| 24 | Colorectal |  |
| 25 | Rectal |  |
| 26 | Colonic |  |
| 27 | Bowel |  |
| 28 | Intestinal |  |
| 29 | 24 OR 25 OR 26 OR 27 OR 28 |  |
| 30 | Surgery |  |
| 31 | Resection |  |
| 32 | 30 OR 31 |  |
| 33 | 29 AND 32 |  |
| 34 | 23 OR 33 |  |
| 35 | 11 AND 34 |  |
| 36 | [REMOVE DUPLICATES] |  |
| 37 | [LIMIT TO ENGLISH LANGUAGE ONLY] |  |
| 38 | [REMOVE NON-ELIGIBLE PUBLICATION TYPES-ABSTRACT, REVIEW, EDITORIAL] |  |
|  | = 4209 |  |

**Supplementary Table 1. Search strategy**

**Supplementary Table 2. Study characteristics of included clinical studies**

| **Study (Year)** | **Country** | **Journal** | **Population** | **Sample size** | **Intervention** | **Microbiome assessment** | | | **Primary outcome** |
| --- | --- | --- | --- | --- | --- | --- | --- | --- | --- |
|  |  |  |  |  |  | **Sample Type** | **Collection Timepoints** | **Analysis Type** |  |
| Zukauskaite (2024)^7^ | Lithuania | British Journal of Surgery | Left-sided resection;  cancer | 40 | Oral mechanical bowel preparation | Stool | Pre-op,  POD 6, 30 | 16S rRNA | Beta diversity (gut microbiota) |
| Sadahiro (2014)^8^ | Japan | Surgery | Left- and right- sided resections;  cancer | 310 | Oral antibiotics | Stool | Pre-op,  POD 7, 14 | 16S rRNA;  c. diff toxin detection | Surgical site infection |
| Reuvers (2023)^9^ | Netherlands | European Journal of Surgical Oncology | Left- and right- sided resections;  cancer | 455 | Selective decontamination of the digestive tract (SDD) | Rectal swab | POD 0 | 16S rRNA | Anastomotic leak |
| Park  (2020)^10^ | Korea | Journal of Clinical Medicine | Anterior resection;  cancer | 60 | Probiotic supplementation | Stool | Pre-op,  POD 21, 28 | 16S rRNA | Anterior resection syndrome |
| Liu  (2010)^11^ | China | Alimentary Pharmacology and Therapeutics | Left- and right- sided resections;  cancer | 100 | Probiotic supplementation | Stool | Pre-op,  POD 3, 10 | 16S rRNA; bacterial culture | Not stated |
| Mizuta (2016)^12^ | Japan | Bioscience | Left- and right- sided resections;  cancer | 60 | Probiotic supplementation | Stool | Pre-op,  POD 7 | 16S rRNA | Not stated |
| Komatsu (2015)^13^ | Japan | Surgery Today | Left- and right- sided resections; mixed | 379 | Synbiotic supplementation | Stool | Pre-op,  POD 7 | 16S rRNA,  faecal organic acid detection | Infective complications |
| Reddy  (2007)^14^ | United Kingdom | British Journal of Surgery | Left- and right- sided resections; mixed | 88 | Synbiotic supplementation | Stool | POD 0 | 16S rRNA | Prevalence of Enterobacteriacae in gut flora |
| Lee (2023)^15^ | Korea | Annals of Surgery | Left- and right- sided resections; cancer | 176 | Arginine and omega-3 fatty acid supplementation | Stool | Not stated | 16S rRNA | Infectious complications |

**Supplementary Table 3. Study characteristics of included pre-clinical studies**

| **Study (Year)** | **Country** | **Journal** | **Animal model** | | | **Intervention** | **Microbiome assessment** | | | **Anastomotic healing assessment** | |
| --- | --- | --- | --- | --- | --- | --- | --- | --- | --- | --- | --- |
|  |  |  | **Animal** | **Site** | **Additional factors** |  | **Sample Type** | **Collection Timepoint** | **Analysis Type** | **Type** | **Timepoint** |
| Boatman  (2024)^16^ | USA | Scientific Reports | Mouse | Distal colon | None | - High-fat/ High-sugar diet - Mechanical bowel preparation & oral antibiotics | Stool | Baseline, POD 0, 4, 7 | - 16S rRNA - SCFA quantification | Macroscopic | POD 7 |
| Guo  (2024)^17^ | Canada | The American Journal of Surgery | Mouse | Distal colon | None | - High-fat/low-fibre diet - Subcutaneous antibiotics | Tissue, Stool | POD 7 | - 16S rRNA - Culture - Collagen quantification | Macroscopic | POD 7 |
| Hyoju  (2021)^18^ | USA | British Journal of Surgery | Mouse | Distal colon | None | Low-fat/ High-fibre dietary prehabilitation | Tissue, Stool | POD 1, 3, 7, 13, 16, 24, 28 | - 16S rRNA - Collagen degradation assay | Macroscopic | POD 1, 3, 7, 13, 16, 24, 28 |
| Liu  (2009)^19^ | Sweden | Colorectal Disease | Rat | Distal colon | Pelvic radiation | Probiotic supplementation | Tissue | POD 4, 7, 11 | - Culture | Histological | POD 4, 7, 11 |
| Castilho (2023)^20^ | Brazil | Surgery Open Science | Rat | Distal colon | None | Probiotic supplementation | Stool | POD 0, 5 | - 16S rRNA | Histological  Mechanical | POD 5 |
| Hajjar  (2021)^21^ | Canada | Clinical Nutrition | Mouse | Proximal colon | None | Prebiotic supplementation | Tissue, Stool | POD 6 | - SCFA quantification - Collagen degradation assay | MacroscopicHistological | POD 6 |
| Mocanu (2021)^22^ | Canada | Nutrients | Mouse | Proximal colon | None | Prebiotic supplementation | Stool | Baseline, POD 0, 7, 14, 28 | - 16S rRNA - SCFA quantification | Histological | POD 28 |
| Wiegerinck (2018)^23^ | USA | British Journal of Surgery | Mouse | Distal colon | *E. faecalis* | Phosphate supplementation | Stool | POD 7 | - Culture - Collagen degradation assay | Macroscopic | POD 7 |
| Hyoju  (2019)^24^ | USA | Annals of Surgery | Mouse | Distal colon | *S. marcescens*, *P. aeruginosa* | Phosphate supplementation | Tissue, Stool | POD 10 | - Culture - Collagen degradation assay | Macroscopic | POD 10 |
| Olivas  (2012)^25^ | USA | PLoS One | Rat | Distal colon | *P. aeruginosa* | Phosphate enema | Tissue, Stool | POD 6 | - Culture - Collagen degradation assay | MacroscopicHistological | POD 6 |
| Jacobson (2020)^26^ | USA | American Journal of Physiology | Mouse | Distal colon | *E. faecalis*,  *P. aeruginosa* | Tranexamic acid enema | Tissue, Stool | POD 8 | - Plasmin activity assay - Flow cytometry - Collagen degradation assay | Macroscopic | POD 8 |
| Jacobson  (2021)^27^ | USA | Annals of Surgery | Mouse | Distal colon | *E. faecalis,*  *P. aeruginosa* | Tranexamic acid enema | Tissue,  Stool | POD 3, 8 | - Plasmin activity assay - Flow cytometry - Collagen degradation assay | Macroscopic | POD 8 |
| Shogan (2016)^5^ | USA | Science Translational Medicine | Rat | Distal colon | Ischaemia | Antibiotic enema | Tissue, Stool | POD 6 | - 16S rRNA, - Culture - MMP9/collagen degradation assay | Macroscopic | POD 6 |
| Jin  (2022)^28^ | China | Journal of Gastroenterology and Hepatology | Rat | Distal colon | None | Faecal microbiota transplantation | Tissue, Stool | POD 0, 7 | - 16S rRNA - RNA-seq | Macroscopic | POD 7 |
| Boatman (2023)^29^ | USA | Gut Microbes | Mouse | Distal colon | None | - High-fat/ High-sugar diet - Faecal microbiota transplantation | Stool | Baseline, POD 0, 4, 7 | 16S rRNA | Macroscopic | POD 7 |
| Hajjar (2023)^30^ | Canada | Gut | Mouse | Distal colon | None | Faecal microbiota transplantation | Stool | POD 0,  POD 6 | 16S rRNA | Macroscopic | POD 6 |
| Shakhsheer (2016)^31^ | USA | Journal of Gastrointestinal Surgery | Rat | Distal colon | None | Morphine | Tissue | POD 0,  POD 6 | - Culture - Collagen degradation assay | Macroscopic | POD 6 |
| Gaines (2020)^32^ | USA | Journal of Gastrointestinal Surgery | Mouse | Distal colon | None | Infliximab | Tissue | POD 7 | - 16S rRNA - Culture | Histological | POD 7 |
